# ^18^F-FDG uptake in PET/CT is a potential predictive biomarker of response to anti-PD-1 antibody therapy in non-small cell lung cancer

**DOI:** 10.1038/s41598-019-50079-2

**Published:** 2019-09-16

**Authors:** Kazuki Takada, Gouji Toyokawa, Yasuto Yoneshima, Kentaro Tanaka, Isamu Okamoto, Mototsugu Shimokawa, Sho Wakasu, Akira Haro, Atsushi Osoegawa, Tetsuzo Tagawa, Yoshinao Oda, Yoichi Nakanishi, Masaki Mori

**Affiliations:** 1 Department of Surgery and Science, Graduate School of Medical Sciences, Kyushu University, 3-1-1 Maidashi, Higashi-ku, Fukuoka 812-8582 Japan; 2 Department of Thoracic Surgery, National Kyushu Medical Center, 1-8-1 Jigyohama, Chuo-ku, Fukuoka 810-8563 Japan; 3 Research Institute for Diseases of the Chest, Graduate School of Medical Sciences, Kyushu University, 3-1-1 Maidashi, Higashi-ku, Fukuoka 812-8582 Japan; 4 Clinical Research Institute, National Kyushu Cancer Center, 3-1-1 Notame, Minami-ku, Fukuoka 811-1395 Japan; 5 Department of Anatomic Pathology, Graduate School of Medical Sciences, Kyushu University, 3-1-1 Maidashi, Higashi-ku, Fukuoka 812-8582 Japan

**Keywords:** Predictive markers, Non-small-cell lung cancer, Tumour biomarkers

## Abstract

To examine the association between ^18^F-fluorodeoxyglucose (^18^F-FDG) uptake in positron emission tomography/computed tomography (PET/CT) and the response to anti-programmed cell death-1 (PD-1) monoclonal antibody therapy in non-small cell lung cancer (NSCLC) patients, 89 patients with advanced or recurrent NSCLC were retrospectively analysed. Maximum standardized uptake value (SUVmax) in ^18^F-FDG PET/CT and the response to anti-PD-1 antibodies were recorded. A cut-off value of SUVmax was determined by receiver operating characteristic curve analysis for patient stratification. Among the 89 patients evaluated, 24 were classified as responders (all partial response), and 65 as non-responders. The average SUVmax of the responders was 15.60 (range, 6.44–51.10), which was significantly higher than that of the non-responders (11.61; range, 2.13–32.75; *P* = 0.0168, Student’s *t-*test). The cut-off SUVmax value selected for stratification was 11.16 (sensitivity and specificity, 0.792 and 0.585, respectively). The response rate of patients with SUVmax value ≥ 11.16 (41.3% [19/46]) was significantly higher than that of patients with SUVmax < 11.16 (11.6% [5/43], *P* = 0.0012, Chi-squared test). The SUVmax in ^18^F-FDG PET/CT is a potential predictive marker of response to anti-PD-1 antibody therapy in NSCLC patients. Further prospective studies of large populations are necessary to validate these results.

## Introduction

The interaction between programmed cell death-1 (PD-1), expressed on activated T lymphocytes, and programmed cell death-ligand 1 (PD-L1), expressed on antigen-presenting cells and tumour cells, has a major role in suppression of the anti-tumour immune response^1^. As such, monoclonal antibodies (mAbs) against PD-1 (e.g. nivolumab and pembrolizumab) or PD-L1 (e.g. atezolizumab) have become one of the standard treatments for patients with advanced or recurrent non-small cell lung cancer (NSCLC). Detection of PD-L1 expression in tumour samples is routinely conducted by immunohistochemistry (IHC) before initiation of treatment with anti-PD-1 or anti-PD-L1. However, expression of PD-L1 alone does not fully predict the response to anti-PD-1 mAbs, and more accurate and convenient response markers are urgently required.

^18^F-Fluorodeoxyglucose positron emission tomography/computed tomography (^18^F-FDG PET/CT) is an essential imaging modality for lung cancer^2,3^ and the majority of patients undergo ^18^F-FDG PET/CT before treatment initiation. Several recent studies have shown that ^18^F-FDG uptake is significantly associated with tumour PD-L1 expression in NSCLC patients^4–6^. However, these studies examined patients with surgically resectable NSCLC, not those with advanced or recurrent cancer; thus, it is unknown whether the relationship between ^18^F-FDG uptake and the efficacy of anti-PD-1 mAbs is also true in more advanced disease.

In this translational study, we evaluated the relationship between ^18^F-FDG uptake on PET/CT and the response to anti-PD-1 mAbs in patients with advanced or recurrent NSCLC patients by dichotomizing the cohort according to the maximum standardized uptake value (SUVmax) on imaging.

## Results

### Patient characteristics

Table 1 shows the characteristics of the 89 patients enrolled in this study. The median age was 67 years (range, 36–88 years); 75 (84.3%) patients were male, and 73 (82.0%) were smokers. *EGFR* or *ALK* gene mutation status was available for 75 (84.3%) patients, and PD-L1 expression data were available for 49 (55.1%) patients. The median SUVmax was 11.40 (range, 2.13–51.10).

**Table 1:** Clinicopathological characteristics of all NSCLC patients.

Characteristic		Value or *n* (%) of patients
Age (years)	Median	67
	Range	36–88
Sex	Female	14 (15.7%)
	Male	75 (84.3%)
ECOG PS	0	18 (20.2%)
	1	63 (70.8%)
	2	7 (7.9%)
	3	1 (1.1%)
Line of treatment	First	17 (19.1%)
	Second	40 (44.9%)
	Third or higher	32 (36.0%)
Smoking history	Never-smoker	16 (18.0%)
	Ex-smoker	39 (43.8%)
	Current smoker	34 (38.2%)
History of radiation therapy	No	58 (65.2%)
	Yes	31 (34.8%)
Clinical stage	IIIB	14 (15.7%)
	IV	52 (58.4%)
	Recurrent	23 (25.9%)
Mutation status (*EGFR* or *ALK*)	Wild type	66 (74.2%)
	Mutated^a^	9 (10.1%)
	Unknown	14 (15.7%)
Histology	Adenocarcinoma	59 (66.3%)
	Squamous cell carcinoma	23 (25.8%)
	Other or unknown^b^	7 (7.9%)
Immune checkpoint inhibitor therapy	Nivolumab	60 (67.4%)
	Pembrolizumab	29 (32.6%)
PD-L1 (22C3) TPS	<1%	11 (12.4%)
	≥1% and <50%	16 (18.0%)
	≥50%	22 (24.7%)
	Unknown	40 (44.9%)
SUVmax	Median	11.40
	Range	2.13–51.10

^a^Nine patients positive for mutant *EGFR*.

^b^Four patients with sarcomatoid carcinoma and three patients with NOS (not-otherwise specified).

*ALK*, anaplastic lymphoma kinase; ECOG PS, Eastern Cooperative Oncology Group performance status; *EGFR*, epidermal growth factor receptor; PD-L1, programmed cell death-ligand 1; TPS, tumour proportion score; SUVmax, maximum standardized uptake value.

### Association between SUVmax and tumour response in NSCLC patients treated with anti-PD-1 mAbs

**Figure 1:**
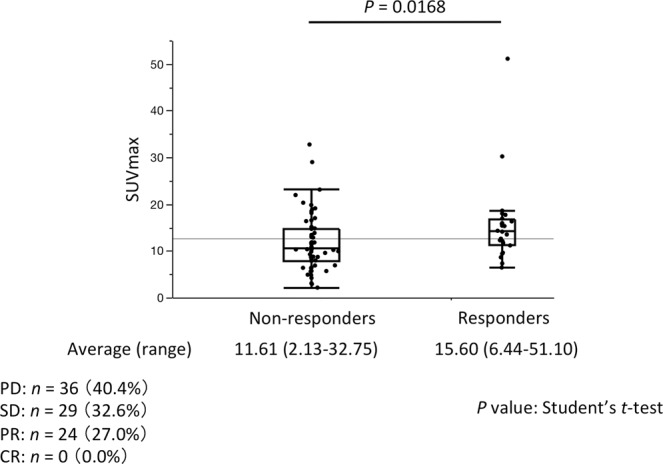
SUVmax of non-responders and responders to anti-PD-1 mAb therapy. Box and whisker plot showing SUVmax of 89 NSCLC patients classified as responders (PR, *n* = 24) and non-responders (PD + SD, *n* = 65). The midline, box edges, and outer bars indicate the median, first and third quartiles, and the upper and lower whiskers, respectively. Dots represent individual patients. SUVmax, maximum standardized uptake value; PD-1, programmed cell death-1; mAb, monoclonal antibody; NSCLC, non-small cell lung cancer; PD, progressive disease; SD, stable disease; PR, partial response; CR, complete response.

Of the 89 patients, 24 were classified as responders (all partial response [PR]) and 65 were non-responders (progressive disease [PD], *n* = 36; stable disease [SD], *n* = 29) following treatment with nivolumab or pembrolizumab. The mean SUVmax was significantly higher for the responders (15.60; range, 6.44–51.10) than the non-responders (11.61; range, 2.13–32.75; *P* = 0.0168, Student’s *t*-test) (Fig. 1).

### Association between SUVmax and response rate in NSCLC patients treated with anti-PD-1 mAbs

**Figure 2:**
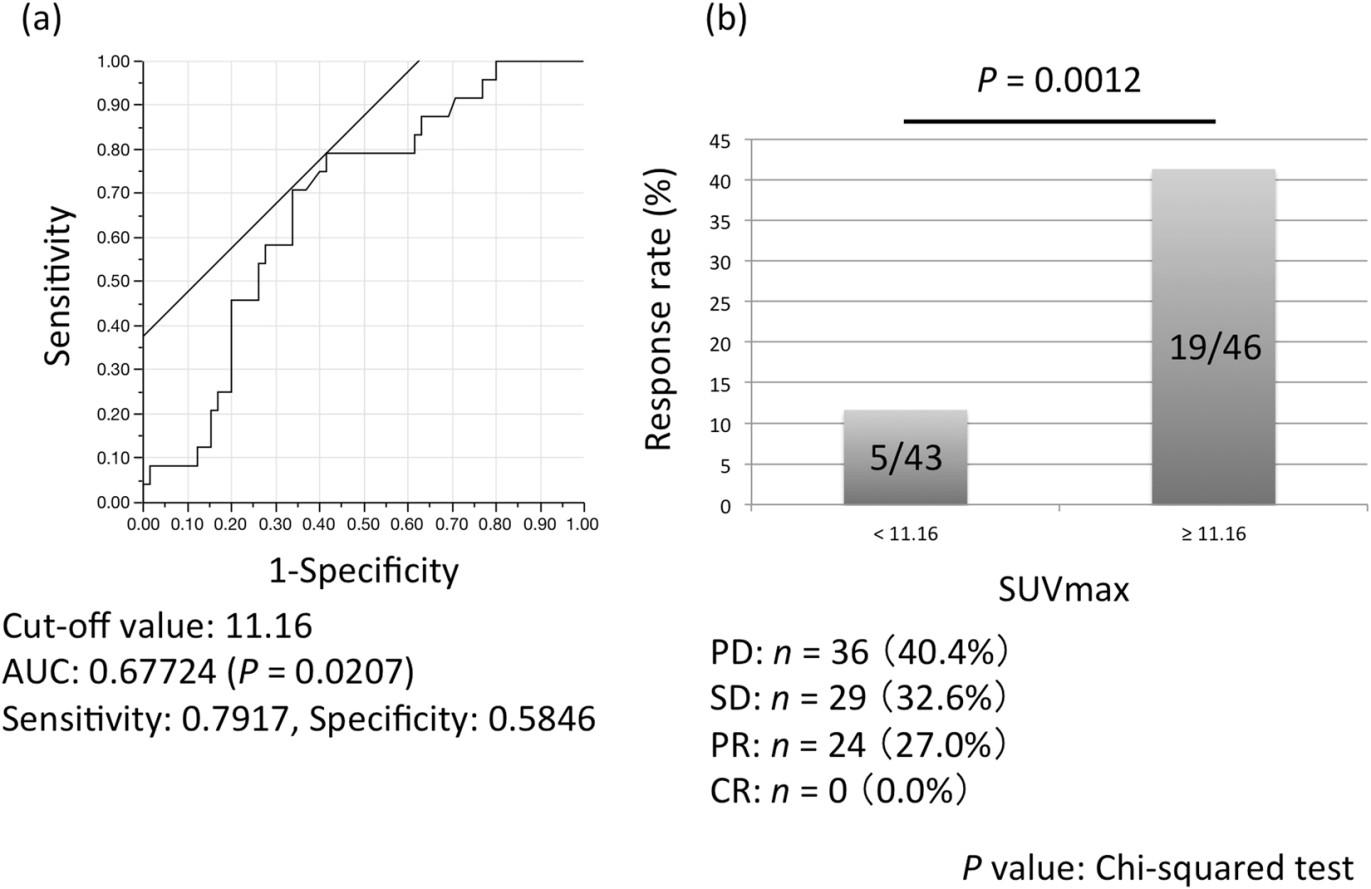
Response rates stratified by SUVmax. (**a**) Receiver operating characteristic curve to determine the optimal cut-off value for SUVmax. (**b**) Average response rates of patients with SUVmax values above and below the cut-off. SUVmax, maximum standardized uptake value; AUC, area under curve; PD, progressive disease; SD, stable disease; PR, partial response; CR, complete response.

To evaluate the relationship between SUVmax and response rate, we selected the optimal SUVmax cut-off value of 11.16 (area under the curve 0.6772, *P* = 0.0207) by receiver operating characteristic curve analysis (Fig. 2a). The response rate of the patients with SUVmax ≥11.16 was significantly higher than that of the patients with SUVmax <11.16 (41.3% [19/46] vs. 11.6% [5/43]; *P* = 0.0012, Chi-squared test) (Fig. 2b).

We conducted univariate and multivariate analyses of the relationship between tumour response and patient characteristics, and high SUVmax (≥11.16) was an independent predictor for tumour response (complete response [CR] or PR) (Table 2).

**Table 2:** Univariate and multivariate analyses of the relationship between tumour response and patient characteristics.

Factors		Univariate analysis		Multivariate analysis	
		OR (95%CI)	*P* value	OR (95%CI)	*P* value
Age (years)	≥67/<67	0.58 (0.22–1.48)	0.2529		
Sex	Male/Female	1.43 (0.36–5.63)	0.6124		
ECOG PS	0 or 1/2 or 3	2.78 (0.32–23.84)	0.3521		
Line of treatment	First or second/third or higher	0.91 (0.35–2.41)	0.8536		
Smoking history	Smoker/never-smoker	1.13 (0.33–3.92)	0.8449		
History of radiation	Yes/no	0.53 (0.19–1.52)	0.2408		
Clinical stage	Recurrent/IIIB or IV	0.48 (0.15–1.61)	0.2359		
Mutation status (*EGFR* or *ALK*)	Wild type/others	2.07 (0.62–6.85)	0.2359		
Histology	SCC/non-SCC	0.48 (0.15–1.61)	0.2359		
SUVmax	≥11.16/<11.16	5.35 (1.78–16.09)	0.0029	5.35 (1.78–16.09)	0.0029

*ALK*, anaplastic lymphoma kinase; CI, confidence interval; ECOG PS, Eastern Cooperative Oncology Group performance status; *EGFR*, epidermal growth factor receptor; OR, odds ratio; SCC, squamous cell carcinoma; SUVmax, maximum standardized uptake value.

### Association between SUVmax and survival of NSCLC patients treated with anti-PD-1 mAbs

**Figure 3:**
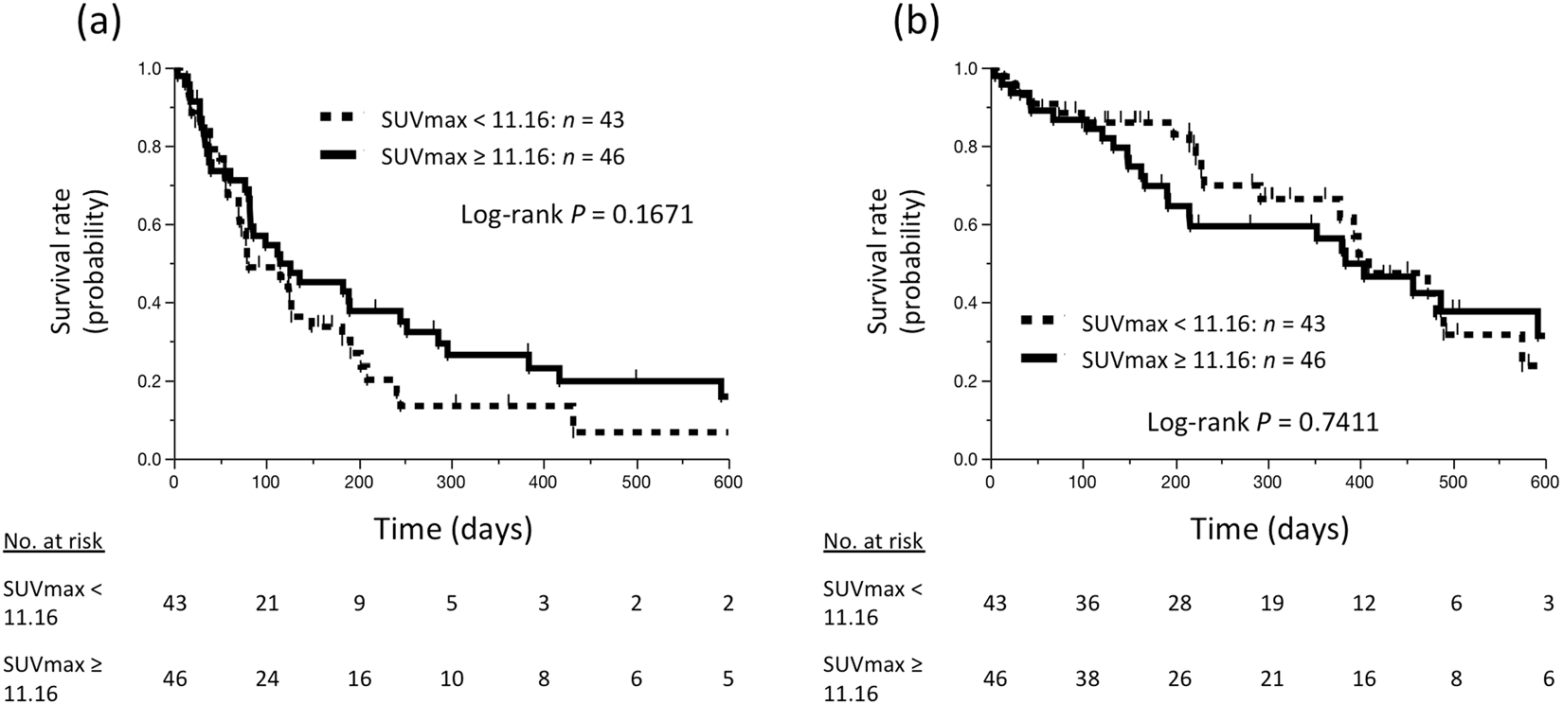
Survival of NSCLC patients stratified by SUVmax. Kaplan–Meier survival curves of progression-free survival (**a**) and overall survival (**b**) of patients stratified by SUVmax. SUVmax, maximum standardized uptake value; NSCLC, non-small cell lung cancer.

The median follow-up time of the study population was 225 days (range, 5–932). Analysis of patient survival using the Kaplan–Meier method revealed that patients with SUVmax <11.16 tended to have shorter PFS than the patients with SUVmax ≥11.16, although the difference did not reach statistical significance (*P* = 0.1671, log-rank test; Fig. 3a). However, no comparable trend was observed for OS (*P* = 0.7411, log-rank test; Fig. 3b).

### Characteristics of the study population stratified by SUVmax

Table 3 shows the features of patients with SUVmax <11.16 and ≥11.16. Of the 49 patients for whom data on tumour PD-L1 expression were available, patients with SUVmax ≥11.16 tended to have higher PD-L1 expression than patients with SUVmax <11.16, although the difference was not significant (*P* = 0.3350, Chi-squared test).

**Table 3:** Characteristics of NSCLC patients stratified by SUVmax.

Characteristic		*n* (%)	SUVmax, *n* (%)		*P* value
			<11.16	≥11.16	
Age (years)	**<**67	43 (48.3%)	20 (46.5%)	23 (50.0%)	0.7421
	≥67	46 (51.7%)	23 (53.5%)	23 (50.0%)	
Sex	Female	14 (15.7%)	9 (20.9%)	5 (10.9%)	0.1907
	Male	75 (84.3%)	34 (79.1%)	41 (89.1%)	
ECOG PS	0 or 1	81 (91.0%)	38 (88.4%)	43 (93.5%)	0.3984
	2 or 3	8 (9.0%)	5 (11.6%)	3 (6.5%)	
Line of treatment	First or second	57 (64.0%)	27 (62.8%)	30 (65.2%)	0.8116
	Third or higher	32 (36.0%)	16 (37.2%)	16 (34.8%)	
Smoking history	Never-smoker	16 (18.0%)	10 (23.3%)	6 (13.0%)	0.2085
	Smoker	73 (82.0%)	33 (76.7%)	40 (87.0%)	
History of radiation	No	58 (65.2%)	28 (65.1%)	30 (65.2%)	0.9920
	Yes	31 (34.8%)	15 (34.9%)	16 (34.8%)	
Clinical stage	IIIB or IV	66 (74.2%)	30 (69.8%)	36 (78.3%)	0.3601
	Recurrent	23 (25.8%)	13 (30.2%)	10 (21.7%)	
Mutation status (*EGFR* or *ALK*)	Wild type	66 (74.2%)	32 (74.4%)	34 (73.9%)	0.9566
	Others	23 (25.8%)	11 (25.6%)	12 (26.1%)	
Histology	Non-SCC	66 (74.2%)	29 (67.4%)	37 (80.4%)	0.1608
	SCC	23 (25.8%)	14 (32.6%)	9 (19.6%)	
PD-L1 (22C3) TPS^a^	**<**50%	27 (55.1%)	16 (61.5%)	11 (47.8%)	0.3350
	≥50%	22 (44.9%)	10 (38.5%)	12 (52.2%)	

^a^For the 49 cases with available data.

*ALK*, anaplastic lymphoma kinase; ECOG PS, Eastern Cooperative Oncology Group performance status; *EGFR*, epidermal growth factor receptor; PD-L1, programmed cell death-ligand 1; TPS, tumour proportion score; SCC, squamous cell carcinoma; SUVmax, maximum standardized uptake value.

## Discussion

In this study, we evaluated the relationship between ^18^F-FDG uptake on PET/CT and the response to anti-PD-1 therapy in patients with advanced or recurrent NSCLC. We found that high SUVmax was significantly associated with better response to anti-PD-1 mAbs. Consistent with this, patients with high SUVmax also showed a trend towards higher tumour expression of PD-L1, although this association was not statistically significant. Nevertheless, the results of this study suggest that ^18^F-FDG PET/CT, which is a relatively non-invasive procedure, might be a useful tool to predict the efficacy of anti-PD-1 mAbs in patients with advanced or recurrent NSCLC.

Our study showed a trend, albeit not significant, towards longer PFS for patients with high SUVmax, which is also consistent with the positive correlation between SUVmax and response rate. However, no such difference was observed for OS. Previous work has shown that FDG uptake by lung cancer cells is regulated by hypoxia, angiogenesis, glucose metabolism, and mammalian target of rapamycin (mTOR) signalling^7^. Thus, tumours with high FDG uptake might have more active AKT–mTOR pathway signalling, resulting in higher proliferative activity of these tumour cells. These findings may provide an explanation for the observation that patients with high SUVmax have longer PFS, but not OS, than patients with low SUVmax. However, many factors influence the survival of lung cancer patients, including driver oncogene status, PD-L1 expression levels, immunological and nutritional status, and treatment after immunotherapy. Therefore, the mechanisms by which SUVmax may be related to the survival of patients with advanced or recurrent NSCLC are unclear.

The rate of ^18^F-FDG uptake in PET/CT could also reflect the metabolic state of the tumour microenvironment. FDG can be taken up by many tumour-associated immune cells, such as tumour-infiltrating lymphocytes (TILs) and tumour-associated macrophages^8,9^. Indeed, FDG uptake in PET/CT has been reported to correlate with the expression of immune-related markers in NSCLC patients^10^. In that study, the authors found a significant association between SUVmax and SUVmean and the abundance of CD8^+^ and PD-1^+^ TILs. Thus, it is possible that the positive correlation between SUVmax and response rate observed in the present study may be related to the number of TILs. However, the previous study was conducted in patients with resectable disease, and not in those with advanced or recurrent cancer. Therefore, whether a similar correlation between FDG uptake and TIL abundance occurs in patients with advanced or recurrent cancer awaits further study. Moreover, FDG is a not specific enough tracer to characterise the presence of an antigen. However, PET/CT is an essential imaging modality for lung cancer, and most lung cancer patients undergo PET/CT before treatment initiation of new drugs. Therefore, the data obtained from PET/CT are easily available in the clinical setting. It is true that more specific tracers now exist such as radiolabelled anti-PD-1 or anti-PD-L1^11–14^, but their accessibility is limited. Hence, these tracers require further study in the future.

The current study has several limitations. First, this was a single-institution retrospective study with a small sample size. However, this is the first report to show a relationship between ^18^F-FDG uptake in PET/CT and the efficacy of anti-PD-1 mAbs in NSCLC patients. Second, a definitive cut-off value for SUVmax has yet to be established and our results should be validated in further prospective studies of larger patient populations. Third, PD-L1 expression data were available for only 49 (55.1%) patients, which may have been insufficient to obtain robust data on the association between PD-L1 expression and SUVmax. Moreover, we conducted univariate and multivariate analyses of the relationship between tumour response and patient characteristics, and high SUVmax was an independent predictor for tumour response (CR or PR). However, we excluded PD-L1 expression data from the analyses because the data were available for only 49 (55.1%) patients. We should conduct the same analyses with PD-L1 expression data in a sufficient sample size in future studies.

In conclusion, the SUVmax in ^18^F-FDG PET/CT obtained at the time of treatment initiation may be important for predicting the efficacy of anti-PD-1 mAbs in NSCLC patients. Consideration of ^18^F-FDG SUVmax and tumour expression of PD-L1 in combination could be a more effective marker of the response to this targeted therapy than the current use of PD-L1 expression alone.

## Methods

### Patients

We retrospectively identified 89 patients with advanced (stage IIIB to IV) or recurrent NSCLC who were treated with anti-PD-1 mAbs (nivolumab or pembrolizumab) between January 2016 and August 2018 at Kyushu University Hospital in Japan. All patients underwent ^18^F-FDG PET/CT before treatment initiation. Anti-PD-1 therapy was administered intravenously at a dose of 3 mg/kg every 2 weeks (nivolumab) or at a fixed dose of 200 mg every 3 weeks (pembrolizumab).

Clinical information and follow-up data were obtained from medical records. The clinicopathological features examined were: age at the time of treatment initiation, sex, Eastern Cooperative Oncology Group performance status, treatment, smoking history, radiation therapy history, clinical stage (7th edition)^15^, driver oncogene status (epidermal growth factor receptor [*EGFR*] and anaplastic lymphoma kinase [*ALK*]), histology, PD-L1 expression status, and SUVmax in ^18^F-FDG PET/CT. *EGFR* status in tumour tissue was determined using the peptide nucleic acid-locked nucleic acid polymerase chain reaction clamp method (Mitsubishi Chemical Medience, Tokyo, Japan)^16^. *ALK* status was assessed by fluorescence *in situ* hybridisation of tumour tissue sections using Vysis ALK Break Apart FISH Probe Kit (Abbott Molecular, Des Plaines, IL, USA)^17^. PD-L1 IHC was performed using clone 22C3 pharmDx antibody (Agilent/Dako, Carpinteria, CA, USA) according to the manufacturer’s recommended methods^18^. In patients with multiple lesions, the highest recorded SUVmax was used for the analysis. Tumour response was assessed by CT every 6 to 8 weeks according to the Response Evaluation Criteria in Solid Tumours (RECIST), version 1.1^19^. According to RECIST criteria, we defined patients with CR or PR as ‘responders’ and patients with SD or PD as ‘non-responders’ in this study. The end of the follow-up period was 30 September 2018. This study was approved by the institutional review board of Kyushu University and was conducted in accordance with the Declaration of Helsinki. This research was defined as a study with human samples by the Japanese guidelines presented by the Ministry of Health, Labour, and Welfare. All methods were performed in accordance with the relevant guidelines. All patients provided written informed consent.

### ^18^F-FDG PET/CT

After fasting for at least 4 h, each patient was intravenously administered 4 MBq/kg ^18^F-FDG. One hour later, scans were conducted from the middle of the thigh to the top of the skull. Images were obtained using an integrated PET/CT scanner (Discovery STE; GE Medical Systems, Milwaukee, WI, USA) or Biograph mCT (Siemens Medical Solutions, Erlangen, Germany). All emission scans were performed in three-dimensional mode, and the acquisition time per bed position was 3 min for Discovery STE and 2 min for Biograph mCT. PET images were reconstructed using the ordered-subset expectation–maximization method (VUE Point Plus) with two full iterations of 28 subsets for the Discovery STE, and iterative True-X algorithm and TOF (Ultra HD-PET) with two full iterations of 21 subsets for the Biograph mCT. The True-X algorithm incorporates an additional specific correction for the point-spread function. The full width at half-maximum values of the Discovery STE and Biograph mCT were 5.2 and 4.4 mm, respectively. A low-dose 16-slice CT (tube voltage 120 kV; effective tube current 30–250 mA, Discovery STE) and a low-dose 32-slice CT (tube voltage 120 kV; use of angular and longitudinal dose modulation, CAREDose4D®, Biograph mCT) from the vertex to the proximal thigh were performed for attenuation correction and for determining the precise anatomical location of lesions before acquisition of PET images. CT scans were reconstructed by filtered back projection into 512 × 512 pixel images with a slice thickness of 5 mm to match the PET scan. ^18^F-FDG uptake in lesions was evaluated using SUVmax, which was calculated by the dedicated workstation for each scanner.

### Statistical analysis

Patient demographics and baseline characteristics were summarised using descriptive statistics or contingency tables. Progression-free survival (PFS) was defined as the time from treatment initiation to clinical or radiographic progression or death, and overall survival (OS) was defined as the time from treatment initiation to the date of the last follow-up or death. Survival curves were constructed using the Kaplan–Meier method and analysed with the log-rank test. SUVmax values between non-responders and responders were compared using Student’s *t*-test. The cut-off value for SUVmax was determined by receiver operating characteristic curve analysis. Associations between SUVmax and response rate or patient characteristics were evaluated using a Chi-squared test. A *P* value of <0.05 was considered statistically significant. All statistical analyses were performed using JMP 13.0 (SAS Institute, Cary, NC).

## Data Availability

All data generated or analysed in this study are included in this published article.
